# Multi-pathway targeted therapy of MASH-HCC using miR-22

**DOI:** 10.1186/s13578-025-01352-7

**Published:** 2025-02-14

**Authors:** Ying Hu, Tahereh Setayesh, Dongguang Wei, Trenton Testerman, Yutong Ji, Yu-Jui Yvonne Wan

**Affiliations:** Department of Pathology and Laboratory Medicine, Research Building III, University of California Davis Health, Room 3400B, 4645 2nd Ave, Sacramento, CA 95817 USA

**Keywords:** Liver cancer, Liver, Metabolism, Fibrosis, ECM, Inflammation, Tumor microenvironment

## Abstract

**Background:**

The treatment options for hepatocellular carcinoma (HCC) are limited, and there is no effective drug that can improve long-term survival rates. Complicated cocktails consisting of multiple medications with toxicities are frequently used to treat cancer. The current study addresses these challenges.

**Methods:**

The study uses metabolic dysfunction-associated steatohepatitis (MASH)-HCC and HCC mouse models established by transfecting the livers using myr-AKT1, NRasV12, and Sleeping Beauty transposase. AAV8-miR-22 was delivered to MASH-HCC and HCC to study its preventive and therapeutic effects. Spatial transcriptomic profiling revealed the signaling pathways affected by miR-22 according to histological locations.

**Results:**

miR-22 treatment effectively treated MASH-HCC and HCC. Treating mice with miR-22 before tumor initiation prevented oncogenesis. The promising anti-cancer effects were revealed by reduced tumor load, fibrosis, and splenomegaly, extending the survival time. miR-22 treatment generated anti-tumor immunity. The favorable treatment outcomes were accompanied by a reduction in dendritic cells, T and B cells, and plasma cells, which were expanded inside the tumors of MASH-HCC. In all animal trials, miR-22 improved metabolism and reduced glycolysis inside the tumors. Moreover, miR-22 profoundly inhibited extracellular matrix (ECM) and targeted MET, PDGF, tyrosine kinase signaling, and IGF pathways inside the tumors. Furthermore, the roles of miR-22 in blocking collagen formation and cross-assembly of collagen fibrils could be due to miR-22's effects in inhibiting Rho GTPase pathways, revealed at the tumor margin.

**Conclusion:**

miR-22 generates anti-HCC effects by targeting many critical pathways in liver carcinogenesis in cancer and tumorigenic niches, potentially revolutionizing HCC treatment.

## Introduction

miR-22 can be induced by retinoic acid, short-chain fatty acids, bile acids, and vitamin D3, with known metabolic benefits and anti-cancer properties [1–3]. miR-22 overexpression shows potential promising effects in colon cancer treatment in cell lines and animal models, partly by exporting nuclear receptors RARβ and NUR77 to cytosol and inducing apoptosis [2]. Furthermore, miR-22 suppresses tumor proliferation, tumorigenesis, and metastasis by targeting proteins, including Protein Kinase C Inhibitor Protein-1 YWHAZ, cluster of differentiation 147 (CD147), galectin-1 (LGALS1), cyclin A2 (CCNA2), and specificity protein 1 (SP1) [1, 4–7]. Moreover, miR-22 is a metabolic silencer that inhibits fibroblast growth factor 21 (FGF21) and its receptor FGFR1, leading to inhibiting ERK1/2 signaling [8]. Recent studies revealed the anti-cancer effects of miR-22 in orthotopic hepatocellular carcinoma (HCC) models [9, 10]. Additional miR-22 targets, including hypoxia-inducible factor 1α (HIF1α), have been uncovered [9].

Carcinogenesis is a complex, multi-step process involving numerous pathways, and effective cancer treatment requires multiple drugs to target these pathways, which can lead to toxic side effects. However, developing a single drug, such as miR-22, that is capable of selectively targeting multiple cancer-specific pathways would represent a groundbreaking advancement in oncology. To explore this potential, it is essential to identify the pathways targeted by miR-22 and understand how it disrupts the underlying mechanisms driving cancer progression. Thus, the main objective of this study is to use miR-22 intercepting HCC at different times and use different HCC models to comprehensively understand miR-22 targeted signaling in the tumor and tumor environment.

HCC commonly arises from chronic liver disease, and metabolic dysfunction-associated steatohepatitis (MASH) is a contributor. Influenced by genetic mutations and environmental factors, MASH can progress to MASH-HCC [11]. We established a MASH-HCC model to monitor the progression of steatosis, inflammation, and tumor immunity [12]. MASH-HCC has an immunosuppressive microenvironment with enhanced inflammation and fibrosis [13]. Thus, another goal of this study is to learn if miR-22 treats MASH-HCC and the spatial impacts of miR-22 in HCC and MASH-HCC.

Our novel findings showed that miR-22 effectively treated MASH-HCC by reducing tumor burden and fibrosis. miR-22 has profound metabolic benefits inside the tumors by improving lipid and bile acid metabolism and inhibiting glycolysis. It also inhibited Rho GTPase signaling and extracellular matrix remodeling. Furthermore, treating mice with miR-22 before tumor initiation prevented oncogenesis and extended the survival time of HCC mice. The data generated using different animal models and intercepted HCC at different time points comprehensively revealed the signaling pathways targeted by miR-22 in tumor and tumor microenvironments.

## Results

### miR-22 treats MASH-HCC

**Fig. 1:**
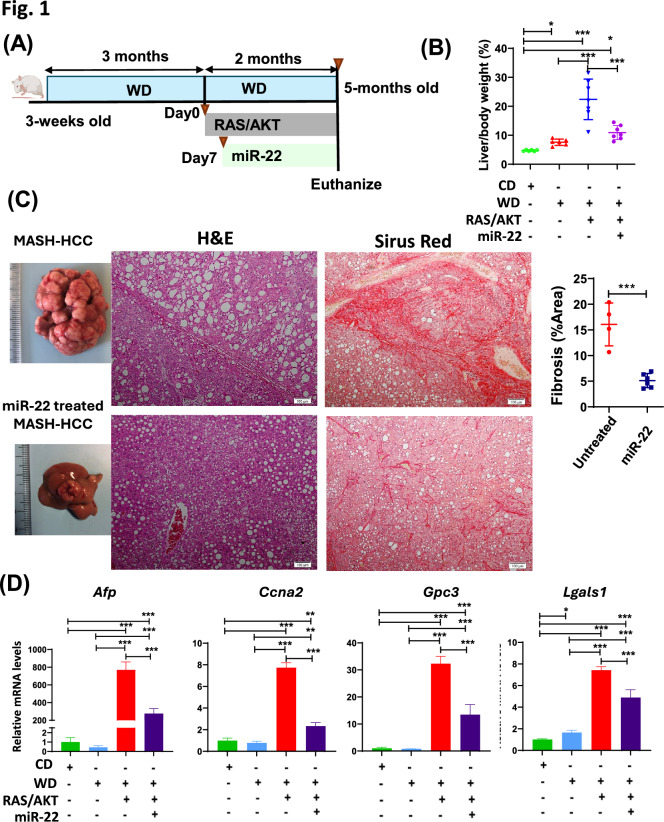
miR-22 treats MASH-HCC. **A** Study design for miR-22 treatment in MASH/HCC. Three-week-old male mice were fed a Western Diet (WD) for 3 months, followed by hydrodynamic injection of Myr-AKT1 and NRasV12 (AKT/RAS) with Sleeping Beauty transposase. 1 week later, AAV8-miR-22 or AAV8 blank control (5 × 10^12^ GC/kg) was administered intravenously. Mice were maintained on a WD during the entire experiment. **B** liver-to-body weight (L/B) ratio, **C** representative liver morphology, H&E, and Sirus red-stained liver sections. Fibrosis was quantified in Sirius Red-stained liver sections and analyzed using ImageJ software. Images were converted to 8-bit grayscale, and a consistent threshold was applied to quantify collagen-positive areas as a percentage of the total tissue area. Results were averaged across fields for each sample. **D** hepatic mRNA levels of *Afp*, *Ccna2*, *Gpc3, and Lgals1*. Data represent mean ± SD (n = 6–8/group). *p* < 0.05, ∗ *p* < 0.01, ∗ ∗ *p* < 0.001 by one-way ANOVA

Our novel data showed that miR-22 treats MASH-HCC, i.e., HCC arose in a steatotic tumor-supporting environment. miR-22 reduced the liver-to-body weight ratio by 50% (from 22 to 11%), which was not significantly different from the WD-fed mice without cancer (Fig. 1A, B). In MASH-HCC, the tumor occupies 90% of the liver section, surrounded by steatosis and fibrosis, as shown by Sirus Red staining. In contrast, miR-22 treatment markedly reduced tumor burden and fibrosis (Fig. 1C). Additionally, miR-22 treatment reduced the expression of α-fetoprotein (*Afp*), cyclin A2 (*Ccna2*), glypican-3 (*Gpc3*), and galectin-1 (*Lgals1*) mRNA (Fig. 1D).

### The spatial effects of miR-22 in altering immune profiles in MASH-HCC

**Fig. 2:**
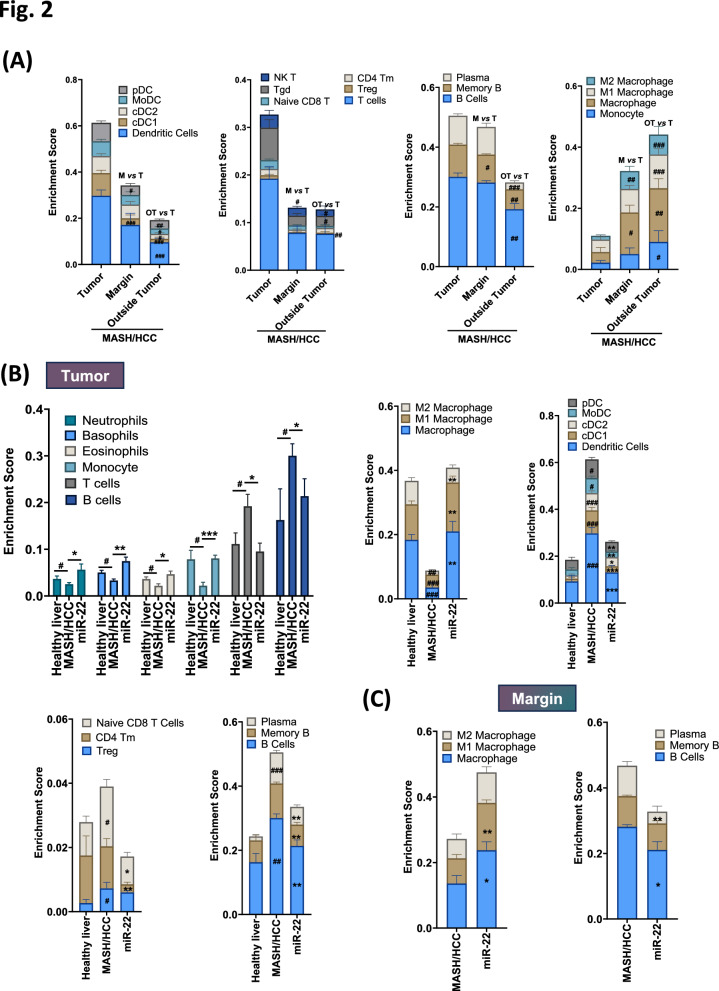
Spatial immune profiles of healthy livers and MASH-HCC treated with or without miR-22. **A** Spatial immune profiling of CD45^+^ cells, including macrophage, dendritic cells (cDC1, cDC2, moDCs, pDCs), T cells (Treg, CD4 Tm, Naïve CD8, Tgd, NK T), B cells, and plasma cells in the tumor, at the tumor margin, and outside the tumor of MASH-HCC mice. # indicates statistical significance based on location comparisons between margin (M) *vs*. tumor (T) or outside tumor (OT) *vs*. tumor (T). **B** Changes in immune cell types, including neutrophils, basophils, eosinophils, monocytes, T cells, and B cells, in the tumor of MASH-HCC in response to miR-22 treatment. **C** Changes in specific immune cell subtypes at the margin regions of MASH-HCC following miR-22 treatment. Data are presented as mean ± SD, analyzed using ANOVA with Tukey's test. * or # *p* = 0.05, ** or ## *p* = 0.01, *** or ### *p* = 0.001, where # indicates comparisons between MASH-HCC vs. healthy liver and * indicates miR-22 treated vs. untreated MASH-HCC

Given the significant impact of a WD in inflammatory signaling [14], we studied the immune profiles of MASH-HCC based on histological location using transcriptomic data. Many immune cells, including dendritic cells, T cells, B cells, and plasma cells, were abundantly found inside the tumors compared to the tumor margin and non-tumor areas. Among the dendritic cells, tumors had more plasmacytoid dendritic cells (pDCs), monocyte-derived dendritic cells (moDCs), and conventional dendritic cells (cDC1 and cDC2) compared with other locations (Fig. 2A). Moreover, tumors had more natural killer T (NKT) cells, γδ T cells (Tgd), naïve CD8 + T cells, memory CD4 + T cells (CD4 Tm), and regulatory T cells (Tregs) (Fig. 2A). However, monocytes and M1 and M2 macrophages were more prevalent at the tumor margin and non-tumor regions than inside the tumors.

miR-22 treatment increased the abundance of neutrophils, basophils, eosinophils, and monocytes but reduced T and B cells inside the tumors (Fig. 2B). Moreover, miR-22 increased M1 and M2 macrophages and reduced dendritic cells that markedly expanded inside the tumors. Furthermore, miR-22 reduced naïve CD8 + T cells and memory CD4 + T cells (CD4 Tm), memory B cells, and plasma cells inside the tumors (Fig. 2B). At the tumor margin, miR-22 treatment expanded M1 but reduced B cells and plasma cells (Fig. 2C). However, no significant changes were observed in immune cell profiling outside the tumor (data not shown). Taken together, MASH-HCC has distinct immune landscapes, and miR-22-treated mice had similar immune profiles to the healthy livers.

### The spatial effects of miR-22 in shifting signaling pathways in HCC and MASH-HCC

We compared the spatial effects of miR-22 in HCC and MASH-HCC, aiming to catch discover all the pathways that might be targeted by miR-22. The anti-cancer effects of miR-22 have been shown previously [9], and the experimental details are repeated here. However, this is the first time the spatial transcriptomic information is uncovered.

**Fig. 3:**
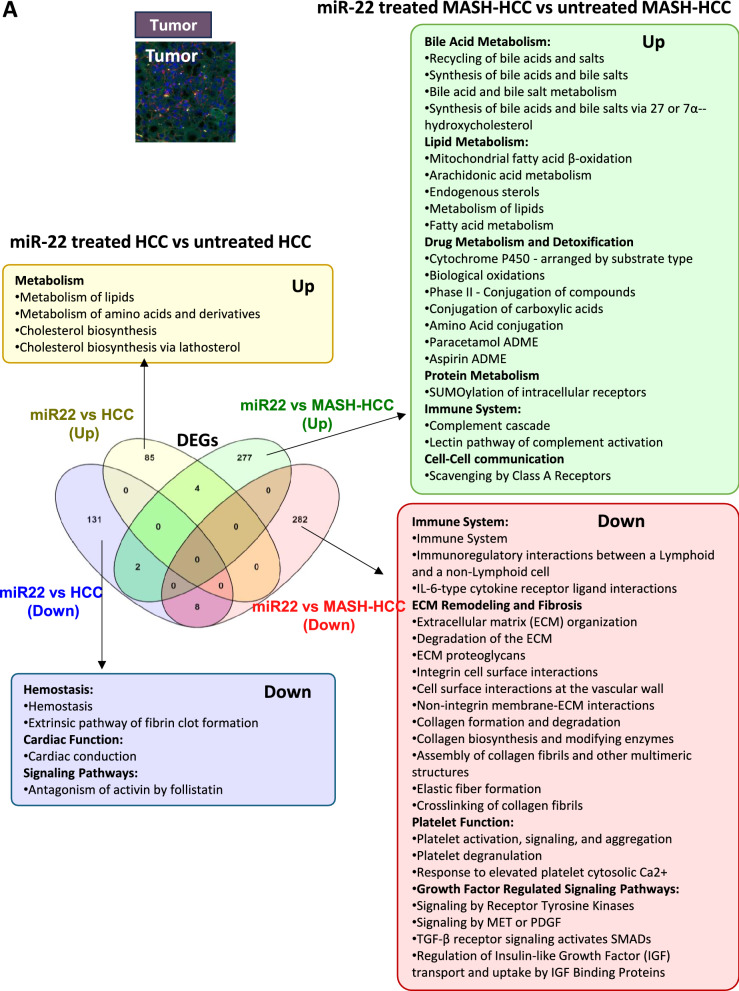

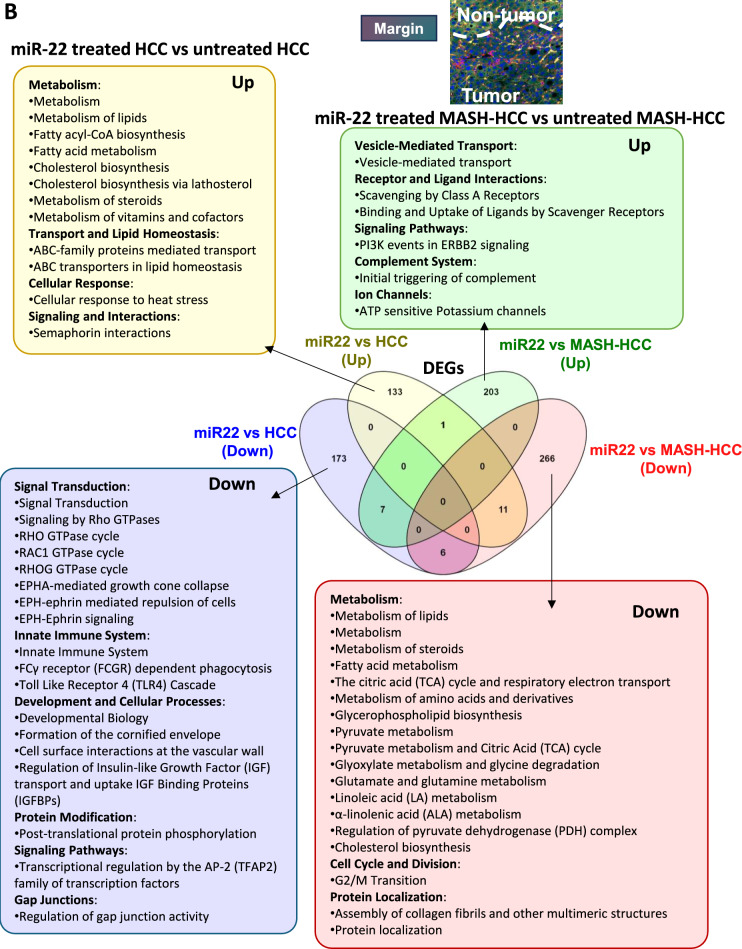

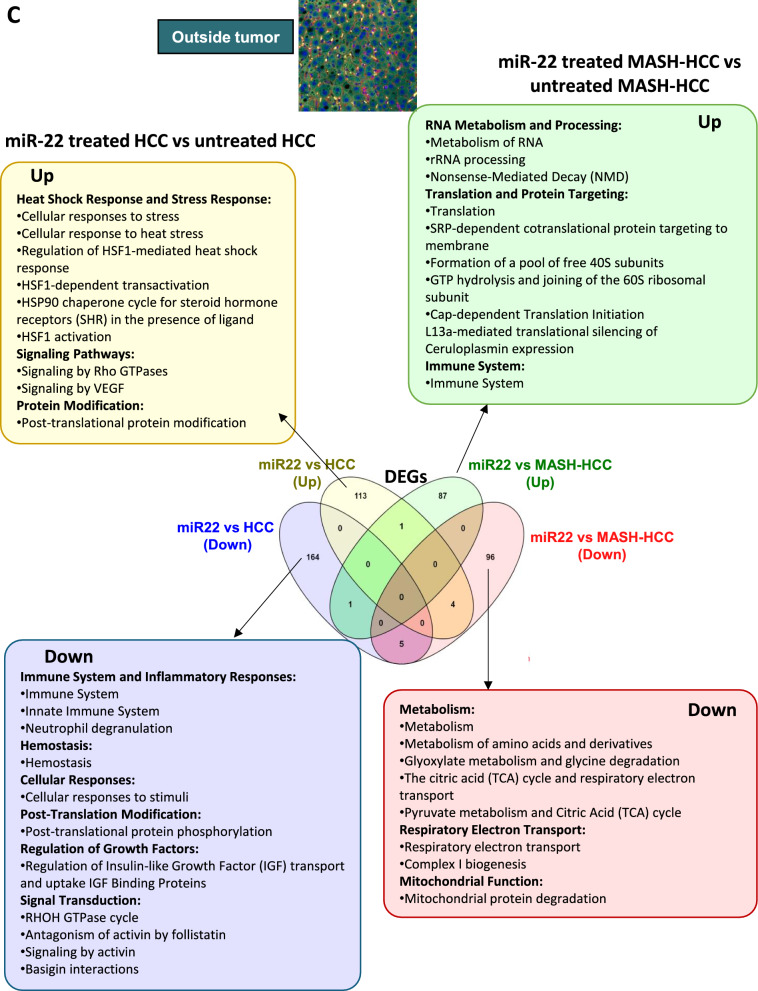
Molecular signatures affected by miR-22 in MASH-HCC and HCC based on the location. Venn diagrams showing expressed gene numbers altered by miR-22 in MASH-HCC and HCC. Pathway analyses were performed using Reactome with significant pathways identified (FDR cutoff: 0.1, minimum fold change of 1.5) across three tissue regions: **A** inside the tumor, **B** at the tumor margin, and **C** outside the tumor areas

Inside the tumors, miR-22 treatment affected many more transcriptomics and pathways in MASH-HCC than in HCC (Fig. 3A). In MASH-HCC, miR-22 upregulated endobiotic metabolism, including bile acid and lipid metabolism, and xenobiotic detoxification. It also enhanced the immune system and cell–cell interaction. Moreover, miR-22 downregulated fibrosis-related pathways, such as ECM organization, integrin cell surface interactions, collagen formation and degradation, collagen fibril crosslinking, and elastic fiber formation. Those changes were accompanied by the inhibition of signaling of tyrosine kinase receptors, MET, TGFβ, and IGF inside the tumors of MASH-HCC (Fig. 3A). Thus, miR-22 had profound impacts on improving metabolism and inhibiting matrix remodeling and tumor growth signaling.

At the tumor margin, the effects of miR-22 in regulating metabolism became complicated. miR-22 improved metabolism in HCC but inhibited it in the MASH-HCC tumor margin, which had a steatotic environment (Fig. 3B). However, miR-22 consistently inhibited growth signaling, including insulin-like growth factor and IGF binding protein factors in HCC and collagen fibril assembly in MASH-HCC (Fig. 3B). Notably, uncovered in the HCC model, miR-22 had profound effects in inhibiting Rho GTPases signaling, which has vital roles in cytoskeleton dynamics affecting cell shape, motility, and migration.

In the nontumor areas, the impacts of miR-22 were distinctly different in HCC, which mostly had normal livers, compared to MASH-HCC, which still had modest steatosis. In the HCC model, miR-22 treatment down-regulated immune and inflammatory signaling. Growth signaling IGF transport and uptake remained inhibited in HCC. In MASH-HCC models, consistent with the findings at the tumor margin, miR-22 treatment down-regulated metabolism, electron transport, and mitochondria function (Fig. 3C). These results show that the roles of miR-22 are context-dependent.

### miR-22 prevents hepatic oncogenesis

**Fig. 4:**
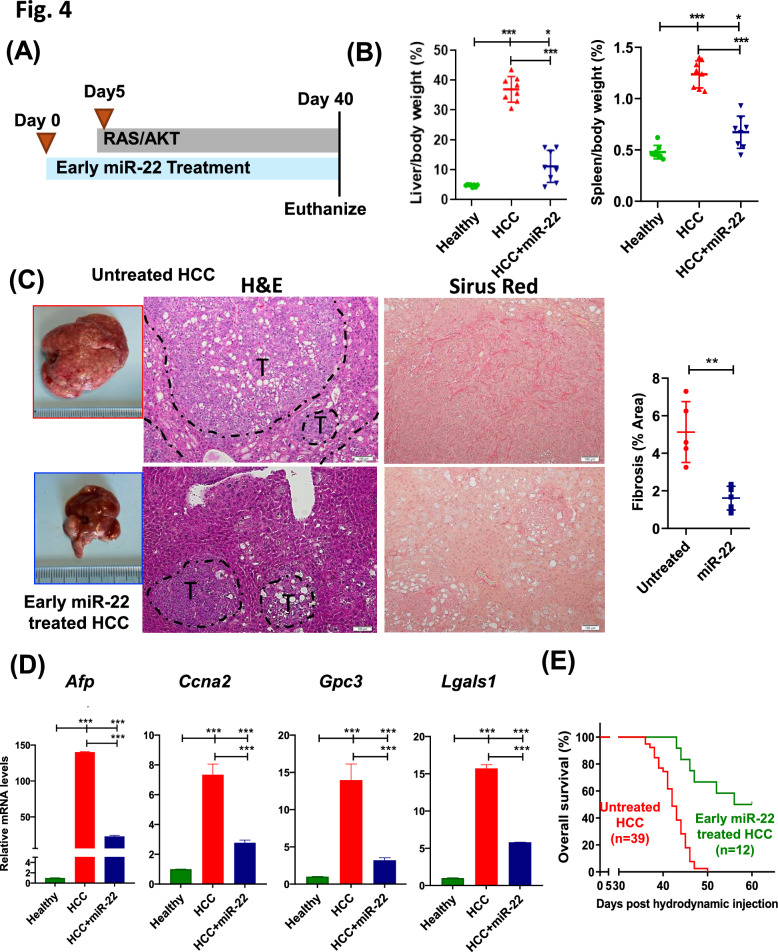
miR-22 prevents liver oncogenesis. **A** A schematic illustration of the study design, **B** liver-to-bodyweight (L/B) ratio, spleen-to-bodyweight ratio, **C** representative liver morphology, H&E-stained liver sections, and Sirus red-stained liver sections. **D** hepatic mRNA levels of *Afp*, *Ccna2*, *Gpc3*, and *Lgals1*. **E** Kaplan–Meier survival curves of overall survival of the studied groups (n = 12–39 mice/group). Data represent mean ± SD (n = 6–8/group). *p* < 0.05, ∗ *p* < 0.01, ∗ ∗ *p* < 0.001 by one-way ANOVA

The anti-cancer effects of miR-22 have been consistently demonstrated in HCC and MASH-HCC models. We further tested whether delivery of miR-22 five days before oncogene injection can have anti-oncogenesis effects. Indeed, early miR-22 treatment reduced the liver/body weight and spleen/body weight ratio (Fig. 4A, B). In control mice receiving AAV8 blank, tumors accounted for more than 90% of liver sections. In contrast, miR-22-treated mice only had small tumors with preneoplastic lesions. Furthermore, early miR-22 treatment markedly decreased fibrosis showing in Sirius Red stained liver sections (Fig. 4C). In addition, early delivery of miR-22 reduced the expression of *Afp*, *Ccna2*, *Gpc3*, and *Lgals1* (Fig. 4D). Furthermore, miR-22 treatment extended the survival time, with a median survival of 58 days in contrast to 42 days for the control mice (Fig. 4E).

### Spatiotemporal effects of early *vs.* late delivery of miR-22 in HCC

The anti-tumor effects should be dynamic depending on the intercepted timing. Thus, we spatially compared the impacts of miR-22 delivery before and after oncogenesis using transcriptomics data.

**Fig. 5:**
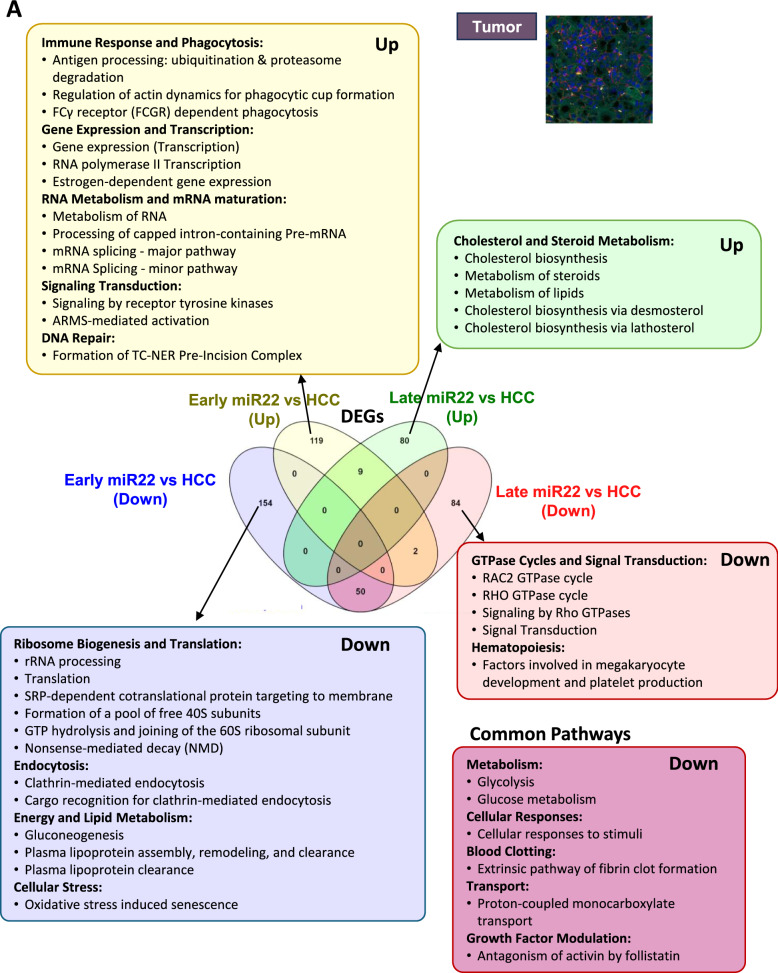

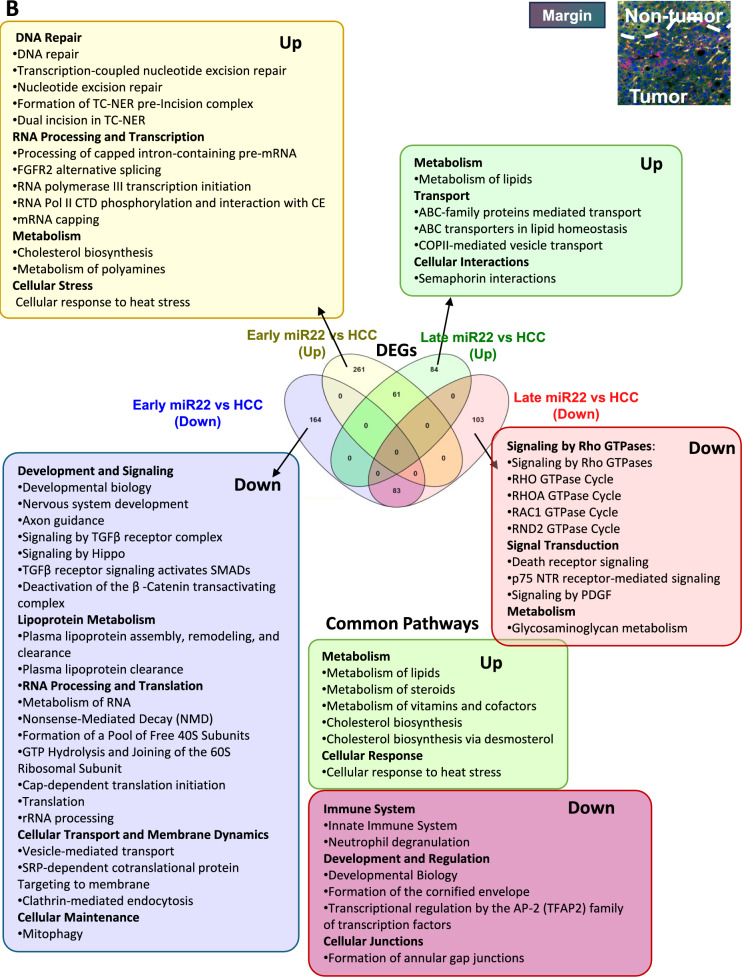

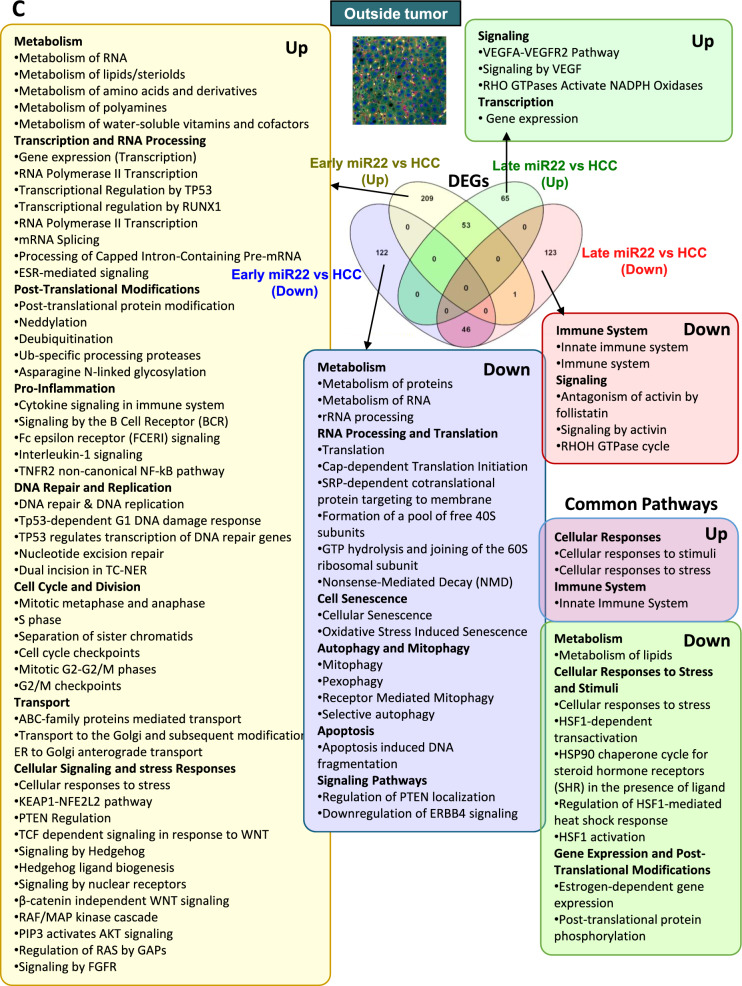
The spatiotemporal effects of miR-22 in HCC. Molecular signatures identified by early and late injection of miR-22 in HCC mice. Venn diagrams showing expressed gene numbers altered by miR-22 treatments. Reactome pathway analysis identified significant pathways (FDR < 0.1, minimum fold change of 1.5) in three locations: **A** inside the tumor, **B** at the tumor margin, **C** in nontumor areas

Early and late interventions within the tumor uncovered distinct and unique pathways affected by miR-22 (Fig. 5A). Early miR-22 treatment showed upregulation in antigen processing, immunity, and phagocytosis. Late treatment uncovered improved cholesterol, steroid metabolism, downregulated GTPase cycles, and hematopoiesis. Both time points showed reduced glycolysis, glucose metabolism, cellular response, blood clotting, and activin antagonism inside the tumors.

At the tumor margin, early miR-22 treatment showed upregulated DNA repair, nucleotide excision repair, RNA processing, cholesterol biosynthesis, and polyamine metabolism, but inhibition in axon guidance, nervous system development, TGFβ, Hippo, and β-catenin signaling. Late miR-22 treatment further revealed upregulated lipid metabolism and ABC-family transport pathways while downregulating Rho GTPase, death receptor, and PDGF signaling. Both treatments showed improved metabolism and inhibition of immune response, consistent with immune profiling data (Fig. 5B).

In nontumor areas, early miR-22 treatment upregulated metabolism, RNA processing, transcription, DNA repair, and replication accompanied by reduced apoptosis, autophagy, and mitophagy. The late treatment only affected a few pathways, including the upregulated VEGFA-VEGFR2 pathway but downregulated Rho GTPase and innate immune pathways. Both treatments upregulated cellular responses to stimuli, suggesting a return to normalcy (Fig. 5C).

### Early miR-22 intervention treats WD-MASH-HCC and HFD-MASH-HCC

**Fig. 6:**
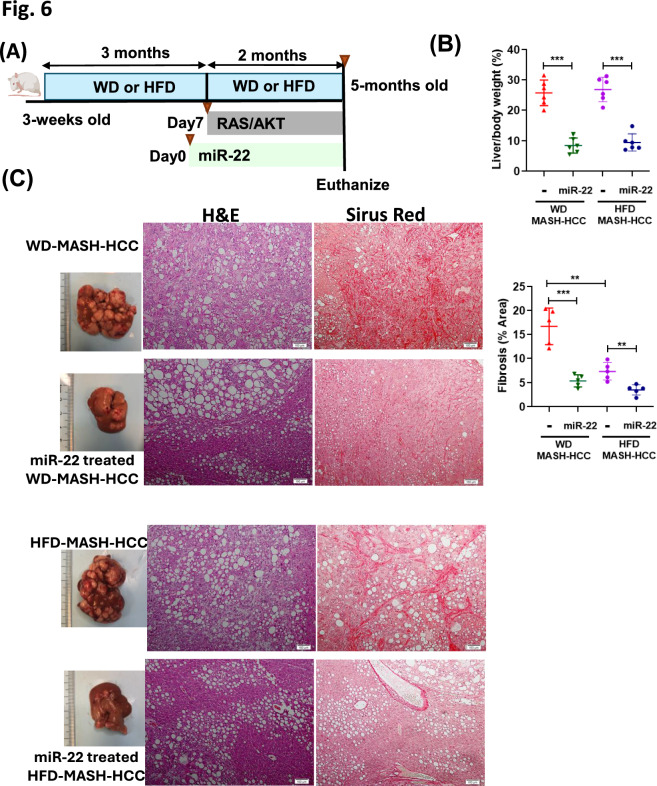
The early intervention with miR-22 effectively treats MASH-HCC in WD- or HFD- induced MASH background. **A** Study design for miR-22 early intervention in MASH/HCC. 3-week-old male mice were fed a Western Diet (WD) or high-fat diet (HFD) for 3 months, followed by hydrodynamic injection of Myr-AKT1 and NRasV12 (AKT/RAS) with Sleeping Beauty transposase. 1 week prior to oncogene delivery, AAV8-miR-22 or AAV8 blank control (5 × 10^12^ GC/kg) was administered intravenously. Mice were maintained on a WD or HFD during the entire experiment. **B** liver-to-body weight (L/B) ratio, **C** representative liver morphology, H&E, and Sirus red-stained liver sections. Data represent mean ± SD (n = 5–6/group). *p *< 0.05, ∗ ∗ *p* < 0.01, ∗ ∗ ∗ *p* < 0.001 by one-way ANOVA

The cancer-preventive effects of miR-22 were further tested in two MASH-HCC models. MASH was induced by either a Western Diet (WD) or High-Fat Diet (HFD) [15]. Early intervention using miR-22 effectively reduced tumor load in both models (Fig. 6). Our novel data demonstrated that delivering miR-22 seven days before oncogene delivery consistently reduced the liver-to-body weight ratio and fibrosis, as evidenced by Sirius Red staining (Fig. 6). Notably, WD-MASH-HCC exhibited more severe fibrosis than HFD-MASH-HCC, suggesting the proinflammatory effect of sucrose (Fig. 6).

## Discussion

This study highlights the promising potential of using miR-22 gene therapy to treat HCC. Demonstrating in different mouse HCC models and intercept oncogenesis at different time points, miR-22 treatment consistently demonstrated anti-HCC benefits. An effective cancer treatment regimen should cover multiple pathways, including targeting growth signaling, apoptosis and cell cycle control, multi-kinase inhibition, and epigenetic modification. In addition, the pathways that support cancer growth and metastasis, including inflammation, epithelial-mesenchymal transition (EMT), and fibrosis, should be intercepted. Our data and others revealed that miR-22 possesses all those characteristics. In addition, its overexpression in HCC mice did not generate noticeable toxicity. Thus, miR-22 could offer a more robust and less toxic treatment option, transforming the current approach to HCC therapy.

Our data also revealed the effectiveness of miR-22 in reducing fibrosis. Moreover, miR-22 halts oncogenesis and offers HCC preventive effects. However, gene therapy is unlikely feasible for cancer prevention; one future direction might be to package miR-22 in different delivery systems, which might be used for advanced fibrosis patients.

miR-22 has impressive immune modulatory impacts. Our previous study uncovered its impacts in reprograming T cells, including reducing IL17-generating T helper cells and expanding cytotoxic T cells. The mechanisms of action are partly due to reduced recruitment of HIF1α, RORγ, and STAT3 to the *Il17a* promoter [9]. The current study utilizing MASH-HCC models revealed that miR-22 promotes the expansion of innate immune cells while reducing adaptive immune cells within tumors. Notably, compared to healthy livers, MASH-HCC tumors exhibited a significant increase in dendritic cell subsets (cDC1, cDC2, pDC, and moDC, Fig. 2B), T regulatory cells (Tregs), and naïve CD8^ +^ T cells. However, miR-22 was found to diminish the presence of these cells in tumors, potentially through mechanisms such as apoptosis, decreased recruitment, or other pathways, which warrant further investigation. Together, MASH-HCC models have pronounced antigen presentations leading to a proinflammatory environment, and miR-22 normalized it.

The anti-HCC effects of miR-22 are closely linked to enhanced metabolic detoxification within tumors. Notably, in the tumor margins and adjacent non-tumorous tissues, miR-22 treatment reduced the metabolism of lipids, amino acids, pyruvate, the TCA cycle, and cholesterol biosynthesis. This reduction may represent an adaptive response to the altered tumor microenvironment. In addition, miR-22 reduces G2/M transition at the tumor margin in the MASH-HCC models. The MASH-HCC has steatosis outside the tumor, thereby having an increased metabolic demand. miR-22 was likely able to reduce the increased demand by inhibiting those metabolic pathways. However, miR-22-treated MASH-HCC remained steatotic, likely due to continuous WD intake. This could be partly due to the effects of miR-22 in silencing metabolic regulators FGF21 and its receptor FGFR1 in steatotic livers, leading to the down-regulation of metabolism-driven growth signaling, i.e., ERK1/2 activation [8]. Thus, miR-22 is a metabolism silencer and tumor suppressor in steatotic livers.

Tumorigenic niches, characterized by hypoxia, inflammation, EMT, and fibrosis, are pivotal in cancer development and progression [16–18]. Continuous feeding of mice with a WD induces chronic inflammation, enhances extracellular matrix (ECM) formation, and promotes EMT [12]. Using the MASH-HCC models, we uncovered the impacts of miR-22 in inhibiting tumorigenic niches. We anticipate that the mechanisms of action could be due to regulating Rho GTPase signaling. Rho GTPases belong to the Ras superfamily of small GTP-binding proteins. While Rho GTPases are not as directly mutated as Ras oncogene in cancers, they play crucial roles in cancer progression, particularly in cell migration, invasion, and metastasis. The most well-known members are RhoA, Rac1, and Cdc42*.* RhoA promotes the formation of stress fibers, actin bundles, and focal adhesions, essential for cell contraction and movement. Rac1 regulates the formation of lamellipodia, broad membrane protrusions involved in cell migration, while Cdc42 governs the formation of filopodia, slender protrusions that help cells sense their environment. [19, 20]. Those processes are critical for immune cell trafficking and cancer metastasis. Rac1 is often overactive in cancers, driving metastasis by enhancing cell motility and invasion [21]. Moreover, Rac1 and Cdc42 are crucial for immune cell migration and antigen presentation [22]. Our data revealed the critical roles of miR-22 in inhibiting Rho, RAC1, RhoG, and RhoH GTPase cycles at the tumor margin or outside the tumors. Those findings might explain the roles of miR-22 in blocking tumorigenic niches.

The anti-tumor effects of miR-22 are dynamics. Euthanizing HCC mice at different time points might generate additional information to uncover the roles of miR-22. In addition, miR-22 targeted genes or pathways in specific cell types remained to be uncovered. For example, the effects of miR-22 in regulating metabolism should be analyzed in immune cells and cancer cells. One study limitation was the delivery system AAV8, which has limited efficiency in getting into immune cells [23]. Although we showed that miR-22 expression was increased in the T cells after miR-22 treatment in the previous study [9], other delivery vehicles should be tested. AAV2 and AAV6 are commonly used. AAV2 has a broad tissue tropism and is one of the most common naturally occurring serotypes found in humans [24]. AAV2 may also engage with the innate immune system via TLR2, as recently demonstrated in nonparenchymal cells, including Kupffer cells and liver sinusoidal endothelial cells, in the human liver [25]. AAV6 has been reported to exhibit higher transduction efficiency than AAV2 in mouse liver cells [26]. Their efficacy in delivery of miR-22 should be tested. Taken together, miR-22 is one drug that targets many cancer pathways, potentially revolutionizing HCC treatment.

## Materials and methods

### Animal models and miR-22 treatment

Male and female FVB/N mice were obtained from Jackson Laboratories (Sacramento, CA) and housed in standard filter-top cages at 22 °C under a 12-h light/dark cycle. Mice received one dose of miR-22 delivered by AAV8 (Applied Biological Materials, Richmond, BC, Canada, 5 × 10^12^ GC/kg intravenously five days before or seven days post oncogene injection. The same dose of AAV8 blank control was used as a control. Oncogene plasmids Myr-AKT1 and NRasV12 (AKT/RAS) (1 µg/g body weight) plus Sleeping Beauty transposase (0.08 µg/g body weight) were delivered by hydrodynamic injection to induce oncogenesis [27, 28].

For the MASH-HCC model, three-week-old male mice were fed a WD or HFD since weaning for 3 months, followed by hydrodynamic delivery of oncogenes [12]. Mice were maintained on the WD (TD. 140,414; Harlan Teklad, Madison, WI) or HFD (TD. 06416; Harlan Teklad, Madison, WI) during the entire experiment. Animal protocols were approved by the Institutional Animal Care and Use Committee (IACUC) at the University of California, Davis (Sacramento, CA, USA).

### Quantification of fibrosis in sirus red-stained liver section

Sirus Red-stained liver sections were imaged and analyzed using ImageJ software [29]. Images were converted to 8-bit grayscale, and a consistent threshold was applied to quantify collagen-positive areas as a percentage of the total tissue area. Results were averaged across fields for each sample.

### RNA isolation and gene expression quantification

Total RNA was extracted using TRIzol Reagent (Thermo Fisher Scientific), and cDNA was generated using a High-Capacity RNA-to-cDNA Kit (Applied Biosystems, Carlsbad, CA, USA). qRT-PCR was performed on a QuantStudio 6 Fast real-time PCR system using Power SYBR Green PCR master mix (Applied Biosystems). Primers were designed using Primer3 Input software version 0.4.0.

### GeoMx® digital spatial profiler (DSP) of transcriptomics analysis

Liver Sections (4 μm) were used for whole transcriptome sequencing via the DSP (NanoString, WA, USA). Morphology markers included CD45, SYTO13 nuclear stain, and Pan-cytokeratin. Slides were stained with RNAscope and GeoMx DSP RNA detection probes per the manufacturer's protocol [12, 27]. RNA targets were associated with GeoMx barcodes. Eight regions of interest (ROIs) per group (4 tumors, 4 margins) were selected. The GeoMx software segmented illumination areas based on biomarkers and UV-cleaved DSP barcodes were collected into a 96-well plate. These barcodes were used for library preparation. Sequenced oligonucleotides were imported into the GeoMx DSP platform for spatial RNA expression analysis.

### Bioinformatics data analysis

The FASTQ sequencing files were converted into digital count files using GeoMx NGS (next gene sequencing) Pipeline software. Quality control and data analysis were performed using the GeoMx DSP Data Analysis suite. Data were filtered by the limit of quantitation and normalized by the third quartile of all counts. Differentially expressed genes (DEGs) were analyzed by linear mixed effect model (LMM) analysis and Benjamini–Hochberg multiple-correction testing with a cutoff log2-transformed fold change > 0.58 and < -0.58 with -log10(*P*) > 1.3.

### Immune cell profiling

The abundance of immune cells was determined by the enrichment score of the expression deviation profile per cell type using single-sample gene set enrichment analysis (ssGSEA) [30, 31]. The obtained enrichment score was normalized, resulting in the ultimate immune cell abundance (http://bioinfo.life.hust.edu.cn/web/ImmuCellAI/).

### Statistical analysis

Statistical analysis was performed using Prism software v8.2.1 (Graph Software). Data were expressed as means ± standard deviation. Statistical significance between two groups was evaluated using a two-tailed Student’s t-test. One-way ANOVA followed by Tukey's t-test was used to compare the statistical differences among multiple groups. Associations were analyzed by linear regression. *p* < 0.05 was considered significant.

## Data Availability

All data of this study are available from the corresponding authors on reasonable request.

## Abbreviations

*Afp*: α-Fetoprotein
*Ccna2*: Cyclin A2
DEGs: Differentially expressed genes
DSP: Digital spatial profiler
EMT: Epithelial-mesenchymal transition
pDCs: Plasmacytoid dendritic cells
moDCs: Monocyte-derived dendritic cells
cDCs: Conventional dendritic cells
ECM: Extracellular matrix
FGF21: Fibroblast growth factor 21
Gpc3: Glypican-3
HCC: Hepatocellular carcinoma
HIF1α: Hypoxia-inducible factor 1α
*Lgals1*: Galectin-1
LMM: Linear mixed effect model
MASH: Metabolic dysfunction-associated steatohepatitis
NKT: Natural killer T
NGS: Next gene sequencing
ROI: Regions of interests
Tregs: Regulatory T cells
